# Use of birth plans by pregnant women and its impact on childbirth: an integrative review

**DOI:** 10.1590/0034-7167-2025-0381

**Published:** 2026-06-22

**Authors:** Paola Coutinho Barreto, Maria Luziene de Sousa Gomes, Roger Rodrigues da Silva, Eremita Val Rafael, Kayo Elmano Costa da Ponte Galvão, José de Ribamar Medeiros Lima, Claudia Teresa Frias Rios, Mônica Oliveira Batista Oriá

**Affiliations:** I Universidade Federal do Maranhão. São Luís, Maranhão, Brazil; II Universidade Federal do Ceará. Fortaleza, Ceará, Brazil

**Keywords:** Obstetric Delivery Planning, Pregnant People, Decision Making, Shared, Humanizing Delivery, Health Promotion., Plan de Parto, Personas Embarazadas, Toma de Decisiones Conjunta, Parto Humanizado, Promoción de la Salud.

## Abstract

**Objectives:**

to assess evidence on the use of birth plans by pregnant women.

**Methods:**

an integrative review, conducted in seven databases without time or language restrictions. The analysis was performed qualitatively and descriptively.

**Results:**

forty-one studies were included. The use of an birth plan: facilitates communication between pregnant women and healthcare professionals; promotes active participation of women; strengthens autonomy; favors shared decision-making; and increases satisfaction with the childbirth process. An association was found with a reduction in cesarean sections, an increase in vaginal deliveries, an increase in Apgar scores, and a greater likelihood of initiating breastfeeding. The most frequent items included skin-to-skin contact, presence of a support person, pain relief methods, expulsive position, information about medical interventions, fluid intake, and ambulation.

**Conclusions:**

the birth plan is an essential tool for humanizing obstetric care and should be presented and discussed during prenatal care.

## RESUMEN

**Objetivos:**

evaluar la evidencia sobre el uso de planes de parto por parte de embarazadas.

**Métodos:**

se realizó una revisión integrativa en siete bases de datos, sin limitaciones de tiempo ni idioma. El análisis fue cualitativo y descriptivo.

**Resultados:**

se incluyeron 41 estudios. El uso del plan de parto facilita la comunicación entre las embarazadas y los profesionales sanitarios; promueve la participación activa de las mujeres; fortalece la autonomía; favorece la toma de decisiones compartida; y aumenta la satisfacción con el parto. Se observó una asociación con una reducción de las cesáreas, un aumento de los partos vaginales, un aumento de las puntuaciones de Apgar y una mayor probabilidad de iniciar la lactancia materna. Los ítems más frecuentes fueron el contacto piel con piel, la presencia de un acompañante, los métodos para aliviar el dolor, la posición para el parto, la información sobre intervenciones médicas, la ingesta de líquidos y la deambulación.

**Conclusiones:**

el plan de parto es una herramienta esencial para humanizar la atención obstétrica y debe presentarse y discutirse durante el período prenatal.

## INTRODUCTION

The use of a birth plan (BP) is recommended by the World Health Organization (WHO)^(1)^, and in Brazil by the Ministry of Health after implementing the Stork Network Program in 2011^(2)^. BP enables the appropriation of information that promotes benefits to women’s autonomy and protagonism in terms of raising awareness among the healthcare professionals who assist them^(3,4)^. In BP, pregnant women express their wishes and desires during their labor (LB), childbirth, and postpartum, which include everything from food and water intake, position at the time of childbirth, use of medications and procedures without real need, guidance on everything that will be carried out^(4)^.

Despite the benefits of developing a BP and its proven effectiveness, lack of awareness of its existence and failure to comply with pregnant women’s expressed wishes remain prevalent. Nurses and physicians should incorporate the use of BP into their consultations, especially in prenatal care provided in Primary Health Care (PHC), to improve care delivery and strengthen communication between pregnant women and the healthcare team in hospital settings. Furthermore, this strategy favors access to information, informed decision-making and the construction of shared responsibility between healthcare professionals and women^(3)^.

BP is within step one of the “*10 passos do Cuidado Obstétrico para Redução da Morbimortalidade Materna*” (10 Steps of Obstetric Care for Reducing Maternal Morbidity and Mortality), which addresses quality encounters centered on the needs of each woman during all contacts with healthcare services, encouraging discussion and joint development of BP^(5)^. It is through BP that the team will learn about pregnant women’s desires and preferences, helping to ensure that these are achieved and respected^(6)^.

In this regard, BP provides women with the opportunity to make choices that value respect, the guarantee of rights, humanized relationships and practices based on scientific evidence^(7)^. Involving women in the decision-making process of their LB through BP can be considered a strategic intervention, which can improve maternal and neonatal outcomes, in addition to providing greater empowerment^(8,9)^.

This study aims to align Sustainable Development Goal 3 (good health and well-being) with the promotion of the use of a BP, encouraging its development and use by women for active participation in childbirth. Therefore, the study is justified for nursing practice by presenting a tool focused on female empowerment and autonomy, in addition to contributing to the dissemination of BP as a means to facilitate the achievement of the following goals: goal 3.1: by 2030, reduce the global maternal mortality rate to less than 70 deaths per 100,000 live births; goal 3.7: ensure universal access to sexual and reproductive healthcare services, including family planning, information and education, as well as the integration of reproductive health into national strategies and programs; and goal 5.6: ensure universal access to sexual and reproductive health and reproductive rights^(10)^.

Previous reviews address aspects of BP such as its purpose, process, impact^(11)^, definitions, content, effects and best practices^(12)^, and its role in shared decision-making^(13)^. No specific review was identified that summarizes evidence on the use of BP by pregnant women and its impacts on the childbirth process. Given this gap, this study aims to assess evidence on the use of BP by pregnant women. As a secondary objective, we sought to analyze the impacts of this use on the care process, the maternal experience, and childbirth outcomes.

## OBJECTIVES

To assess evidence on the use of BP by pregnant women.

## METHODS

### Ethical aspects

This study was based on published and publicly available data; therefore, it does not require submission to or approval from a Research Ethics Committee. It is worth noting that the fundamental ethical principles of scientific research were followed, including methodological transparency, academic integrity, and respect for copyright.

### Study design

This is an integrative literature review, based on the theoretical framework proposed by Whittemore and Knafl, carried out in five stages: guiding question elaboration; primary study search and selection; primary study assessment; data analysis; and review presentation^(14)^. This methodological framework was adopted to ensure a systematic approach to synthesizing evidence. The aim was to obtain a comprehensive understanding of the use of BP by pregnant women and to identify gaps to be explored in future studies^(14)^. The writing of the study followed the Preferred Reporting Items for Systematic Reviews and Meta-Analyses (PRISMA) recommendations^(15)^.

### Guiding question

The guiding question defined to conduct this integrative review was: what does the scientific evidence reveal about the use of BP by pregnant women? To develop this question, the acronym PICo (Population, Interest and Context) was adopted, with P =population (pregnant women), I =interest (BP) and Co =context (healthcare services).

### Eligibility criteria

Primary studies related to the topic, conducted with pregnant women, were included without restriction of language or publication period. A decision was made not to establish a time frame, as the objective of this study was to gather all available literature on the use of BP by pregnant women. This decision is justified because it is the first integrative review to synthesize the evidence on the subject, encompassing everything from the first publications to the end of the data collection period. Course completion papers, dissertations, theses, conference proceedings, and editorials were excluded.

### Study search and selection

The search for primary studies was conducted on September 17, 2024, in the following databases: Medical Literature Analysis and Retrieval System Online (MEDLINE)/PubMed (via the National Library of Medicine), Web of Science, Scopus, Cumulative Index to Nursing and Allied Health Literature (CINAHL from EBSCO), Cochrane, EMBASE (Elsevier), and *Literatura Latino-Americana e do Caribe em Ciências da Saúde* (LILACS) via the Virtual Health Library. The databases were accessed free of charge through the Coordination for the Improvement of Higher Education Personnel Journals Portal.

The search terms selected from the Medical Subject Headings were applied to MEDLINE, Web of Science, Scopus, CINAHL, Cochrane, LILACS, and the Emtree Terms in the Excerpta Medica database (EMBASE). The search strategies were developed by combining descriptors and keywords using the Boolean operators OR and AND, according to Chart 1.

**Chart 1:** Search strategy used in the databases, São Luís, Maranhão, Brazil, 2024

Database	Search strategy
MEDLINE/PubMed Web of Science Scopus CINAHL Cochrane EMBASE LILACS	("Pregnant women” OR Pregnancy OR Women) AND ("Birth plan” OR “Maternity care plan")

*MEDLINE - MEDical Literature Analysis and Retrieval System Online; CINAHL - Cumulative Index to Nursing and Allied Health Literature; EMBASE - Excerpta Medica dataBASE; LILACS - Literatura Latino-Americana e do Caribe em Ciências da Saúde.*

The results identified in the databases were exported to the Rayyan software^(16)^. Study selection was carried out by two reviewers, independently, in two stages and followed the (identification, screening and inclusion) PRISMA flowchart recommendations^(15)^. The first stage involved reading the titles and abstracts and applying eligibility criteria. Subsequently, the reviewers met to discuss any discrepancies in the selection process and reach a consensus. In the next stage, the full texts were read, and eligibility criteria were applied again. Any disagreements at the end of the stage were resolved with the opinion of a third reviewer. It should be noted that the manual search of the reference lists of included primary studies was performed to identify additional evidence related to the topic of interest.

### Data collection

Data collection corresponding to study characterization occurred through the development and use of a data extraction form, extracting the following variables: authorship; year of publication; country; study objective; study design; and main results. This stage was performed independently by two reviewers. In cases where disagreements occurred, a meeting was held to discuss the findings until a consensus was reached.

### Data processing and analysis

The results were analyzed and synthesized descriptively using a narrative synthesis approach. First, the studies were grouped to provide a comprehensive overview of the scope, nature, and distribution of the studies included in the review, presented in a characterization chart. To classify the level of evidence (LoE) of the studies, we used the model proposed by Melnyk and Fineout Overholt^(17)^, which is divided into the following levels: level I - evidence from a systematic review or meta-analysis of all relevant randomized controlled clinical trials or from clinical guidelines based on systematic reviews of randomized controlled clinical trials; level II - evidence derived from at least one well-designed randomized controlled clinical trial; level III - evidence obtained from well-designed clinical trials without randomization; level IV - evidence from well-designed cohort and case-control studies; level V - evidence originating from a systematic review of descriptive and qualitative studies; level VI - evidence derived from a single descriptive or qualitative study; and level VII - evidence derived from the opinions of authorities and/or reports of expert committees. The results were then discussed based on thematic categories identified by the authors after reading the included studies.

## RESULTS

**Figure 1:**
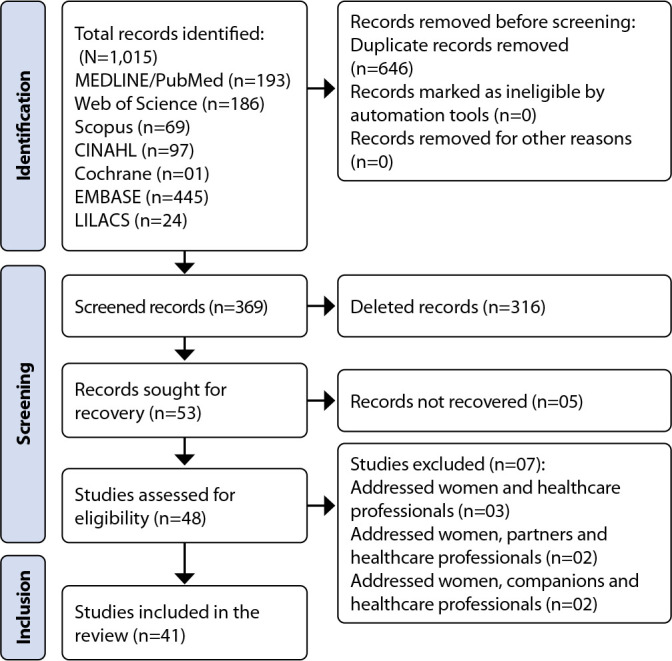
Preferred Reporting Items for Systematic Reviews and Meta-Analyses flowchart: search and selection of included articles, São Luís, Maranhão, Brazil, 2024

In total, 1,015 records were identified. After removing duplicates, 369 remained for screening. After screening titles and abstracts, 316 records were excluded for not meeting the inclusion criteria, and five records could not be retrieved, leaving 48 potentially relevant studies. After full-text review, seven did not meet the criteria, leaving 41 studies (Figure 1).

### Characterization of included studies

Chart 2 presents the descriptive summary of primary studies according to authorship, year of publication, country of study, study design, sample, setting, and LoE.

**Chart 2:** Summary of the studies included in the integrative review (N=41), São Luís, Maranhão, Brazil, 2024

ID	Author, (year) and country	Objective	Study design, sample and setting	LoE
E1	Ahmadpour *et al*. (2024)^(18)^ Iran	Explore and understand the perspectives of women who used a BP during childbirth.	Study: qualitative Sample: n=14 women Setting: Taleghani Hospital in Tabriz, Iran Sector of activity: not specified	VI
E2	Artieta-Pinedo *et al*. (2024)^(19)^ Spain	Explore which of the options included in BPs are of greatest interest to women and which are more controversial.	Study: cross-sectional Sample: n=247 women Setting: 20 midwifery clinics Sector of activity: not specified	VI
E3	Chantry *et al*. (2023)^(20)^ France	Assess the prevalence of postpartum women with preferences for their LB and childbirth, expressed verbally in the delivery room or as a written BP, and to study maternal, obstetric, and organizational factors associated with their expression.	Study: cross-sectional Sample: n=11,633 postpartum women Setting: 513 maternity hospitals in France Sector of activity: not reported	VI
E4	Barnes *et al*. (2023)^(21)^ Australia	Explore the experiences and perspectives of women who used a planned cesarean section in an Australian tertiary maternity hospital.	Study: cross-sectional Sample: n=294 participants Setting: Australian tertiary maternity hospital Sector of activity: not reported	VI
E5	Guo *et al*. (2023)^(22)^ China	Assess the effects of a continuous partnership-based BP on the outcomes and childbirth experience of local women in Haikou, China.	Study: randomized clinical trial Sample: n=90 women Setting: tertiary hospitals in the city of Haikou, Hainan province, China Sector of activity: hospitals’ obstetrics clinic	II
E6	Mohaghegh *et al*. (2023)^(8)^ Iran	Investigate the effect of a BP combined with prenatal preparation classes on maternal and neonatal outcomes.	Study: randomized clinical trial Sample: n=300 women Setting: four public health centers in Tehran, Iran + two private hospitals and one hospital affiliated with the armed forces Sector of activity: not reported	II
E7	Alba-Rodríguez *et al*. (2022)^(23)^ Spain	Determine the impact of presenting a BP on women’s personal experience, focusing on their expectations and the level of satisfaction achieved.	Study: qualitative Sample: n=7 women Setting: nursing room at the tertiary hospital of the Andalusian Public Health System (Spain) Sector of activity: nursing consultation room in a health center	VI
8	Ahmadpour *et al*. (2022)^(24)^ Iran	Investigate the effect of BP on maternal and neonatal outcomes.	Study: randomized clinical trial Sample: n=106 pregnant women Setting: Taleghani Educational Hospital in Tabriz, Iran Sector of activity: Obstetrics Clinic of Taleghani Medical Research and Training Hospital	II
9	López-Gimeno *et al*. (2022)^(25)^ Spain	Identify whether women who presented a BP at the hospital have better obstetric outcomes and are more satisfied with the birthing experience.	Study: randomized clinical trial Sample: n=461 pregnant women Setting: four Primary Care Units of the National Health System of Catalonia (Spain) Sector of activity: not reported	II
10	Trigueiro *et al*. (2022)^(6)^ Brazil	Describe the experience of pregnant women who attended nursing consultations from 37 weeks onwards and who developed their BP.	Study: qualitative Sample: n=19 pregnant women Setting: routine risk maternity care in Curitiba Sector of activity: maternity outpatient clinic	VI
11	Hidalgo-Lopezosa *et al*. (2021)^(26)^ Spain	Compare obstetric and neonatal outcomes between women with and without a BP.	Study: retrospective case-control study Sample: n=457 pregnant women Setting: tertiary hospitals in southern Spain Sector of activity: four public tertiary hospitals with the largest coverage in each province	IV
12	López-Gimeno *et al*. (2021)^(27)^ Spain	Identify the percentage of pregnant women who presented a BP in five hospitals in Spain, the reasons why some women did not do so, and how the presentation of BP relates to obstetric outcomes and selected pain relief methods.	Study: descriptive and multicenter Sample: n=422 women Setting: primary health centers in several health districts in Barcelona (Catalonia, Spain) Sector of activity: five Sexual and Reproductive Health Care units of the Catalan Health Institute in primary care centers located in Catalonia	VI
13	Loiola *et al*. (2020)^(7)^ Brazil	Analyze the perception of women who used a BP in a birthing center in southeastern Brazil.	Study: descriptive study with a qualitative approach Sample: n=11 postpartum women Setting: birthing center in Rio de Janeiro Sector of activity: prenatal care clinics	VI
14	Jolles *et al*. (2019)^(28)^ Netherlands	Assess the rate of BPs, characteristics of women, and postpartum satisfaction scores at a single center in the Netherlands.	Study: retrospective Sample: n=1,159 women Setting: Amalia Children’s Hospital, Radboudumc, Nijmegen Sector of activity: academic hospital	VI
15	Soriano-Vidal *et al*. (2018)^(29)^ Spain	Assess the influence of midwife-led prenatal education classes on women’s preferences regarding childbirth.	Study: multicenter, observational, and prospective Sample: n=212 pregnant women Setting: reference health centers in southeastern Spain Sector of activity: health departments of La Ribera (March to September) and Xàtiva-Ontinyent (January to September)	VI
16	Afshar *et al*. (2018)^(30)^ United States	Examine whether the presence of a BP was associated with the mode of childbirth, obstetric interventions, and patient satisfaction.	Study: prospective cohort Sample: n=300 women Setting: large urban tertiary hospital in Los Angeles Sector of activity: birthing centers and postpartum recovery wards	IV
17	Westergren *et al*. (2019)^(31)^ Sweden	Elicit the perceptions of pregnant women about childbirth, as expressed in their BPS, and, through a feminist lens, analyze their desires, fears, values, and beliefs about childbirth, as well as their expectations regarding their partner and midwife.	Study: qualitative Sample: n=132 women Setting: a medium-sized city in northern Sweden Sector of activity: online: through electronic prenatal and intrapartum medical records	VI
18	Hidalgo-Lopezosa *et al*. (2017)^(32)^ Spain	Determine the degree of compliance with the requests that women include in their BPs and to assess their influence on key obstetric and neonatal outcomes.	Study: retrospective, descriptive and analytical Sample: n=178 women with BP Setting: tertiary level hospital of the Andalusian Public Health System Sector of activity: not specified	VI
19	Divall *et al*. (2017)^(33)^ United Kingdom	Explore women’s opinions about BPs and their experiences with creating and using them.	Study: qualitative Sample: not specified Setting: online Sector of activity: not specified	VI
20	Anderson *et al*. (2017)^(34)^ Hawaii	Describe how a group of culturally and educationally diverse women who presented for obstetric care in Honolulu, Hawaii, rated communication, satisfaction, and trust with the use of a standardized BP during LB and childbirth.	Study: quality improvement project Sample: n=81 women Setting: Kapiʻolani Medical Center for Women & Children in Honolulu, Hawaii Sector of activity: not reported	VI
21	Afshar *et al*. (2017)^(35)^ United States	Determine whether the mode of childbirth differed between women who attended childbirth education classes, had a BP, or both, compared to those who did not attend classes or have a BP.	Study: cross-sectional retrospective Sample: n=14,630 women Setting: urban tertiary hospital in Los Angeles Sector of activity: urban tertiary care center	VI
22	Mouta *et al*. (2017)^(36)^ Brazil	Analyze how BP facilitated women’s empowerment during LB and childbirth.	Study: exploratory qualitative Sample: n=11 postpartum women Setting: birthing center located in the municipality of Rio de Janeiro, Brazil Sector of activity: not reported	VI
23	Mei *et al*. (2016)^(37)^ United States	Categorize individual BP requests and determine whether the number of requests and the fulfillment of those requests are associated with satisfaction with the childbirth experience.	Study: prospective cohort Sample: n=109 women Setting: a large urban tertiary care medical center in Los Angeles Sector of activity: not reported	IV
24	Suárez-Cortés *et al*. (2015)^(38)^ Spain	Understand, analyze, and describe the current situation of BPs and childbirth in the studied context, comparing the LB and childbirth process and its outcome between women who presented a BP and those who did not.	Study: quantitative, cross-sectional, observational, descriptive, and comparative cohort study Sample: n=9,303 women Setting: Virgen de la Arrixaca University Clinical Hospital in Murcia Sector of activity: delivery room	IV
25	Vila-Candel *et al*. (2015)^(39)^ Spain	Verify if there are differences in the preferences reflected by pregnant women in BP before and after receiving maternal education sessions.	Study: epidemiological, observational, longitudinal, and prospective Sample: n=249 pregnant women Setting: La Ribera Health Department of the Valencian Health Agency Sector of activity: primary care centers of the La Ribera Health Department	VI
26	Whitford *et al*. (2014)^(40)^ Scotland	Consider the use of a standard BP section within a national maternity registry maintained by women.	Study: exploratory, qualitative, and longitudinal Sample: n=42 women Setting: two regions of the National Healthcare service Board in northeast Scotland Sector of activity: the interviews were conducted in the women’s homes or on the premises of the university or healthcare service	VI
27	Hidalgo Lopesoza *et al*. (2013)^(41)^ Spain	Determine if BPs are associated with better obstetric and neonatal outcomes.	Study: retrospective case-control study Sample: n=182 women Setting: hospital in Córdoba, Spain Sector of activity: not reported	IV
28	Magoma *et al.* (2013)^(42)^ Tanzania	Determine the effectiveness of BPs in increasing the use of specialized care during childbirth and the postnatal period among participants in prenatal care in a rural district.	Study: cluster randomized trial Sample: n=900 women Setting: Ngorongoro district, Arusha region Sector of activity: 16 health units and, subsequently, women’s homes in the village in the district for the postnatal interview	II
29	Hadar *et al*. (2012)^(43)^ Israel	Determine whether the introduction of a pre-prepared BP on admission to the LB ward has an impact on obstetric outcomes.	Study: retrospective Sample: n=154 women who were compared to a matched control group of 462 women Setting: tertiary medical center in Israel Sector of activity: not reported	VI
30	Pennell *et al*. (2011)^(44)^ United States	Describe the preferences and outcomes related to anesthesia and analgesia of women who used a BP for LB and childbirth.	Study: prospective cohort study Sample: n=63 Setting: tertiary care university hospital in the United States Sector of activity: not reported	IV
31	Sheridan *et al*. (2011)^(45)^ Ireland	Compare BP preferences between Irish and Nigerian pregnant women.	Study: observational study Sample: n=632 women Setting: Unified Maternity Services in Cork, Ireland Sector of activity: prenatal clinic (reserve)	VI
32	Sato; Umeno (2011)^(46)^ Japan	Investigate pregnant women’s knowledge of BPs and to clarify the relationship between postpartum women’s awareness of their BP and their degree of satisfaction with their childbirth experience, as well as to examine the role of the midwife in contributing to childbirth satisfaction.	Study: survey using a questionnaire Sample: n=442 postpartum women Setting: three municipal obstetric clinics Sector of activity: not specified	VI
33	Kuo *et al*. (2010)^(47)^ China	Assess the effects of BPs on women’s fulfillment of expectations regarding childbirth, their control over the childbirth process, and their overall experiences.	Study: randomized, single-blind, controlled clinical trial Sample: n=296 pregnant women Setting: seven hospitals in Taiwan Sector of activity: medical units in northern and central Taiwan	II
34	Yam *et al*. (2007)^(48)^ Mexico	Assess the feasibility and acceptability of introducing a BP in a hospital serving low-socioeconomic status Mexican women and to document the perspectives of women and healthcare professionals on the advantages and barriers in implementing a BP program.	Study: exploratory Sample: n=9 pregnant women Setting: *Hospital de la Familia* in Ciudad Juárez, Mexico Sector of activity: not reported	VI
35	Deering *et al*. (2006)^(49)^ United States	Assess what type of patient prepares a BP, identify common requests made, and determine how strictly they are followed during LB and childbirth.	Study: descriptive Sample: n=67 women Setting: National Naval Medical Center military training hospital (Bethesda, Maryland) Sector of activity: LB and childbirth unit of the National Naval Medical Center	VI
36	Gulbrandsen *et al*. (2004)^(50)^ Norway	Discover whether the use of a BP contributed to providing women with a better experience and safety during pregnancy, childbirth, and motherhood, and to describe the experiences of women in using such a form.	Study: questionnaire-based research Sample: n=300 pregnant women Setting: health centers in the municipalities of Eidsvoll, Fet, Gjerdrum, Rælingen and Ullensaker Sector of activity: Akershus University Hospital	VI
37	Lundgren *et al*. (2003)^(51)^ Sweden	Assess the effects of answering a questionnaire and formulating a BP at the end of pregnancy on women’s childbirth experiences.	Study: questionnaire-based research Sample: n=271 women Setting: prenatal clinics in Sweden Sector of activity: normal childbirth ward and special childbirth ward	VI
38	Brown; Lumley (1998)^(52)^ Australia	Assess the use of a BP and examine the differences in social and obstetric characteristics, and intrapartum experiences of women who used and did not use a BP.	Study: population-based research Sample: n=1,336 women Setting: 127 hospitals in Victoria, Australia Sector of activity: not reported	VI
39	Whitford; Hillan (1998)^(53)^ Scotland	Investigate the use and effects of BPS and how women perceive them.	Study: retrospective Sample: n=143 primigravidas Setting: university hospital with approximately 3,000 births per year in Dundee, Scotland Sector of activity: not reported	VI
40	Moore; Hopper (1995)^(54)^ Australia	Assess the ease of use and effectiveness of introducing BP in two hospitals.	Study: descriptive Sample: n=100 women Setting: in two district hospitals in southwest Sydney (Australia) Sector of activity: hospital clinics for prenatal care	VI
41	Smoleniec; James (1992)^(55)^ England	Test the hypothesis that having a BP for LB was associated with an increased risk of operative childbirth.	Study: descriptive Sample: n=124 women Setting: not reported Sector of activity: not reported	VI

*ID - Identification; LoE - level of evidence; BP - birth plan; LB - labor.*

Concerning time frame, the included studies date from 1992 to 2024. The year 2017 (n=5) concentrated the majority of the analyzed productions. Most of studies were conducted in Spain^(19,23,25-27,29,32,38,39,41)^ and in the United States^(30,35,37,44,49)^. Regarding the methodological approach, the studies were mostly characterized as qualitative^(6,7,18,23,31,33,40)^ followed by clinical trials^(8,22,24,25,42,47)^. The study samples ranged from 247^(19)^ and 14,630 women^(35)^. The settings were diverse, including hospital srtiings^(8,18,20-22,24,26,28,30,32,35,37,38,41,43-45,47-49,51-54)^, primary care^(25,27,29,39,42)^, outpatient clinic^(6,7,19,23,34,46)^, birthing house^(7,36)^ and online^(31,33)^. Regarding LoE, most studies were classified as level VI^(6,7,18-21,23,27-29,31-36,39,40,43,45,46,48-55)^.

The sample for this study, based on the analyzed articles, is shown in a summarized form in the supplementary material. Therefore, the critical analysis and synthesis of the selected studies were carried out qualitatively (Chart 3), resulting in the categorization of seven main themes: BP knowledge; BP use; BP items; effect of BP on maternal outcomes; effect of BP on neonatal outcomes; satisfaction and compliance with BP; and BP in prenatal care.

**Chart 3:** Summary of proponents of birth plan use by pregnant women, São Luís, Maranhão, Brazil, 2024

BP knowledge	Lack of knowledge of BP^(36)^ Does not know or knows it superficially^(6)^ Never heard of a BP before^(48)^
**BP use**	Improves the overall birth experience^(18,44)^ Facilitates communication^(11,13,18,21,23,24,33,34,40,44,47,48,54)^ Promotes women’s active participation during childbirth^(8,18,47)^ Provides physical and mental preparation^(18,52)^ Promotes women’s empowerment and autonomy^(6,7,9,13,36)^ Facilitates shared decision-making^(13)^ and autonomy and control^(11,13,24,33,44,47)^
**BP items**	Skin-to-skin contact^(8,9,19,21,23,25,27,29,38,39,45,48)^ Presence of a companion^(19,31,45)^ or support person^(21)^ Pain relief method using pharmacological and non-pharmacological methods^(7,9,18,19,25,27,44,52)^ Information and control over medical intervention^(19,22,26)^ Expulsive position^(29,38,39,45)^ Walking during labor^(18,49)^ Epidural use^(27,45)^ Fluid intake^(29,38,39)^ Food intake^(38)^ Lowering of the surgical mesh^(20)^
**Effect of BP on maternal outcomes**	Promotes a reduction in cesarean sections and a higher rate of vaginal deliveries^(8,9,18,24,32,34,43,48,49,52)^ Reduces medical intervention^(8,22,26,32,37,40)^ Improves birth outcomes^(21)^ Reduces anxiety^(6,8,22,32,40)^ Reduces fear^(8,13,18,24,51)^ Reduces psychological symptoms of depression and PTSD^(23)^ Optimizes the birth experience^(21)^
**Effect of BP on neonatal outcomes**	Increased neonatal Apgar scores at one minute^(9,24,26,32,44)^ Delayed cord clamping^(38)^ Best results in umbilical cord pH^(9,26,32)^ Greater likelihood of initiating breastfeeding in the delivery room^(8,19,23,27,29,37,39,48)^ Lower propensity to have a NB admitted to the NICU^(30,34,35)^
**Satisfaction and compliance with BP**	Greater satisfaction with the birthing process^(8,9,11,18,24,28,34,46-48)^ Provides a better experience, taking into account women’s preferences^(23,52)^ Support and communication during labor increase women’s satisfaction with childbirth^(24)^ Birth outcomes are generally positive^(9,11,50)^ Allows for freedom and autonomy in childbirth, making them feel active and respected as women in their desires^(7)^ Women with a BP and a spontaneous vaginal delivery more often gave a high score for postpartum satisfaction^(28)^ High compliance with BP decreased the percentage of cesarean sections and their children had better neonatal outcomes^(32)^ Meeting birth expectations ensured a greater degree of control and participation by women^(47)^ Greater requests fulfilled correlated with greater overall satisfaction^(37)^ Women who knew that staff had looked at their BP during labor appreciated that their preferences were followed or at least discussed^(40)^ BP positively influences labor and its completion, increasing the dimensions of safety, effectiveness and satisfaction of women, as well as their empowerment^(39)^
**BP in prenatal care**	Educational tool^(13)^ Influences women’s preferences regarding childbirth^(29,39)^ Greater expression of written or verbal preferences^(20)^ Influences a positive birth experience^(18)^ Women who have had childbirth would encourage other women to use it as well^(54)^ Allows the expression of needs and preferences, increasing trust, and improves communication among the team^(54)^ The nursing consultation enabled the development of BP^(6)^ Promotes women’s participation in decision-making^(8,13)^ Favors individualized obstetric care, ensuring respect for their choices^(13)^ Reduces fear and anxiety^(8,13)^ Enables the acquisition of knowledge^(7)^ Addresses the individual needs of their patients^(42)^

*BP - birth plan; LB - labor; PTSD - post-traumatic stress disorder; NB - newborn; NICU - neonatal intensive care unit.*

## DISCUSSION

The body of evidence analyzed allowed us to understand the factors that influence the use of prenatal care by women in different contexts around the world. The studies highlight notable findings regarding its use, such as facilitating communication, promoting women’s active participation during childbirth, strengthening autonomy and control over their own bodies, as well as fostering shared decision-making and increasing satisfaction with the birth process.

Furthermore, BP is associated with a reduction in the number of cesarean sections, an increase in the rate of vaginal deliveries, a decrease in fear and anxiety, an increase in Apgar scores in the first minute of life, and a greater likelihood of breastfeeding initiation in the delivery room. Another relevant benefit is the reduced propensity of newborns to be admitted to Neonatal Intensive Care Units. Among the most frequent items in BP are skin-to-skin contact, the presence of a companion, the adoption of pain relief methods, choice of the expulsive position, obtaining information and control over medical interventions, as well as the possibility of water intake and ambulation during LB.

BP is among the techniques that should be encouraged during pregnancy, according to international standards recommended by the WHO. However, in some healthcare services that assist pregnant women and women in LB, BP is still little encouraged^(36)^. Despite being a right of pregnant women, BP is still little known or superficially understood by many women in various regions, which negatively impacts its use. A study conducted in Paraná with 13 postpartum women found that 69% of them did not know what BP was, and 77% did not even realize it was a legal right^(4)^. Similarly, in another study carried out in Rio de Janeiro, only one of the eleven postpartum women interviewed reported knowing BP, while all the others denied any familiarity with the instrument^(36)^. Similarly, a survey conducted in a public maternity hospital in Curitiba with 19 pregnant women found that 13 of them were unaware of BP or had only heard about it, without knowing exactly what it was, reaffirming the lack of more in-depth knowledge on the subject^(6)^.

It is clear that only a small portion of women receive information about BP from healthcare professionals. A survey conducted in Curitiba, Paraná, found that other sources, such as support networks and the internet, were the main means of accessing information about BP and topics related to pregnancy and childbirth, highlighting the need for greater engagement by healthcare professionals in disseminating and providing guidance on this instrument^(6)^. Similarly, a study conducted in Andalusia, Spain, revealed that while most women valued prenatal classes, they reported the need for staff to be up-to-date and for classes to be better coordinated. Despite being informed about the existence of prenatal classes, they reported difficulty obtaining detailed information about them, turning to other sources such as the internet, books, associations, family, and friends for guidance^(23)^. These findings reinforce the importance of healthcare professionals being the main interlocutors in BP promotion and clarification, guaranteeing women qualified and reliable access to this tool.

BP, although legally recognized, still has low dissemination among healthcare professionals, hospitals, and maternity wards, which compromises its practical application^(36)^. Few services are able to implement it effectively, and many professionals do not even know its meaning^(3)^. Among the institutional and sociocultural barriers, the lack of knowledge and inadequate understanding of BP by healthcare professionals, the heterogeneity of the plans themselves and the lack of effective communication between pregnant women and the care team stand out, which generates distorted perceptions of choice and limits women’s autonomy^(6,11)^. Pregnant women’s lack of knowledge about the birth process and the birth itself also contributes to doubts, fears, and insecurities^(6)^. Furthermore, plans drawn up without prior discussion with care providers can generate unrealistic expectations and dissatisfaction, especially in highly medicalized obstetric contexts, resulting in a loss of autonomy during childbirth^(11)^.

To overcome these challenges, it is essential that healthcare services adopt strategies that prioritize communication and shared decision-making. BP should be valued as a tool that facilitates dialogue, strengthens women’s autonomy, and promotes more humane obstetric practices^(11,36)^. Collaborative development, with healthcare professionals’ active involvement, is crucial to align expectations, optimize obstetric outcomes, increase satisfaction, and expand pregnant women’s sense of control^(11)^. Hence, the nursing consultation is configured as a strategic space for health education, enabling the use of BP as an effective educational resource to clarify doubts, reduce anxiety, promote the empowerment of pregnant women and their companion and establish a bond with motherhood, in addition to offering clear guidance on LB, childbirth and the postpartum period^(6,36)^.

Additionally, it is recommended to adopt structural and organizational strategies, such as integration of BP into prenatal care protocols, multidisciplinary training focused on woman-centered care, and use of digital tools that facilitate the preparation, recording, and sharing of the document between the pregnant woman and the health team^(3,4,6,11,23)^. Healthcare professionals play a fundamental role in implementing BP, especially nurses, whose work begins in prenatal care, when BP should be presented and its development encouraged. During prenatal care, pregnant women have the opportunity to clarify questions and receive support from nurses in preparing the document, strengthening their understanding of LB and birth and encouraging the expression of their wishes, generally focused on humanizing and experiencing the natural process^(36)^. The development of BP is validated as an important communication tool between pregnant women and healthcare professionals, although it is still necessary to increase team involvement and training so that they are more aware of issues related to respecting women’s choices^(23)^.

A qualitative study conducted in Iran identified prenatal preparation as a key factor for a positive birth experience, highlighting that BP implementation was fundamental in increasing pregnant women’s awareness, promoting physical and mental readiness, and empowering them to make informed choices about safe birth methods^(18)^. Similar findings were observed in a study conducted in Paraná, in which 13 participants reported that prenatal consultations reduced their anxiety and fear, providing greater tranquility, security, and confidence for childbirth, both in relation to physiological aspects and motherhood routines^(6)^. These results reinforce the importance of prenatal education and women’s active involvement in the decision-making process during childbirth. Educational classes and the development of prenatal care are fundamental strategies for preparing pregnant women physically and psychologically, in addition to providing information and alternatives for managing LB^(8)^.

In relation to the items covered in BP, a descriptive study carried out in Spain highlighted that most women consider the presence of their partner during childbirth essential, the maintenance of an intimate environment, continuous contact with the baby from birth and the provision of information with a request for consent before any intervention^(19)^. Additionally, a narrative review found that women with premature LB were more likely to initiate breastfeeding in the delivery room. Although epidural analgesia was the most commonly used method for pain relief, these women were more likely to use combined non-pharmacological methods^(9)^.

Another determining factor in the desire to develop and use a plan is the degree of compliance with its guidelines. A study of 178 women in Andalusia, Spain, revealed that only 37% of pregnant women who submitted a plan had their preferences largely respected. Furthermore, it was observed that the higher the level of compliance with the plan, the better the maternal and neonatal outcomes^(32)^. Among the main reasons for non-compliance with BP, the unpredictability of the birth process stands out, such as the occurrence of dystocia or unexpected events that require changes in the initially planned conduct^(32)^.

While BP represents an important planning and communication tool, it does not guarantee that LB and birth will proceed as planned. This reinforces the importance of managing expectations and promoting clear communication between healthcare professionals and pregnant women about possible variations in the process^(18)^.

For BP to become an effective care tool, its implementation must be discussed with healthcare management and developed with the active involvement of professionals working in these settings. The document must be adapted to local circumstances, thoroughly discussed with pregnant women and their companions, and continually updated, ensuring that it reflects individual needs and provides more positive and safe birth experiences for all involved^(6)^.

### Study limitations

The limitations of this study are related to the fact that some included studies presented weaknesses in their methodological description, such as the lack of specification of the study design, year of completion, and sample size. Furthermore, a predominance of research conducted in the United States and Spain was observed, with little national production on BP. Furthermore, most of included studies correspond to level VI of evidence, predominantly qualitative and descriptive, which implies less methodological robustness and restricts the generalization of the findings to different contexts. Despite these limitations, we sought to carefully follow the recommendations for developing integrative reviews. The strengths of the study include the fact that it is novel, providing information on BP use, with easy search and analysis of content in studies from various countries, through open access on educational platforms with concrete data for its preparation, without financial cost or the need for authorization from the research ethics committee.

### Contributions to nursing, health or public policies

The findings of this review expand knowledge about the use of BP in various regions of the world, supporting the creation of educational materials that can be used in clinical practice and future studies. Furthermore, the results contribute to the dissemination of knowledge among professionals, promoting BP appreciation as a tool for pregnancy-centered care and decision-making. This approach also encourages continued research on the topic, strengthening evidence-based practices in maternal health.

## CONCLUSIONS

The studies analyzed indicate that the use of BP facilitates communication between pregnant women and healthcare professionals, promotes women’s active participation, strengthens their autonomy, and favors shared decision-making, resulting in greater satisfaction with the birth process. Furthermore, BP is associated with better maternal and neonatal outcomes. Among the most frequently reported items by pregnant women are skin-to-skin contact, the presence of a companion, pain relief methods, choice of delivery position, information on medical interventions, water intake, and walking during LB.

The results of this review reinforce that BP is a fundamental tool for promoting more humane obstetric care centered on women’s needs. Its presentation and encouragement during prenatal care are strategic for strengthening pregnant women’s autonomy, improving the care provided, and improving the childbirth experience. This review contributes to deepening knowledge about the use of BP in the global context. We suggest further research to explore the knowledge and use of BP in Brazil, especially in PHC, where prenatal care is provided and pregnant women have their first contact with healthcare services.

## Data Availability

The research data are available within the article.

## References

[1] World Health Oganization (WHO) (2018). WHO recommendations: intrapartum care for a positive childbirth experience.

[2] Ministério da Saúde (BR) (2017). Diretrizes nacionais de assistência ao parto normal: versão resumida.

[3] Narchi NZ, Venâncio KCMP, Ferreira FM, Vieira JR. (2019). Individual birth planning as a teaching-learning strategy for good practices in obstetric care. Rev Esc Enferm USP.

[4] Wiggers C, Araujo M, Martins W, Strada CFO. (2021). Conhecimento das puérperas sobre o plano de parto em um município do oeste do Paraná. Rev Eletrôn Acervo Científ.

[5] Ministério da Saúde (BR) (2022). 10 Passos do Cuidado Obstétrico para Redução da Morbimortalidade Materna.

[6] Trigueiro TH, Arruda KA, Santos SD, Wall ML, Souza SRRK, Lima LS. (2022). Experiência de gestantes na consulta de Enfermagem com a construção do plano de parto. Esc Anna Nery.

[7] Loiola AMR, Alves VH, Vieira BDG, Rodrigues DP, Souza KV, Marchiori GRS. (2020). Plano de parto como tecnologia do cuidado: experiência de puérperas em uma casa de parto. Cogitare Enferm.

[8] Mohaghegh Z, Javadnoori M, Najafian M, Abedi P, Kazemnejad Leyli E, Montazeri S (2023). Effect of birth plans integrated into childbirth preparation classes on maternal and neonatal outcomes of Iranian women: a randomized controlled trial. Front Glob Womens Health.

[9] Kohan S, Hajihashemi M, Valiani M, Beigi M, Mohebbi-Dehnavi Z. (2023). Maternal-infant outcomes of birth planning: a review study. J Educ Health Promot.

[10] World Health Organization (WHO) (2015). Transforming our world: the 2030 Agenda for Sustainable Development.

[11] Bell CH, Muggleton S, Davis DL. (2022). Birth plans: a systematic, integrative review into their purpose, process, and impact. Midwifery.

[12] Ghahremani T, Bailey K, Whittington J, Phillips AM, Spracher BN, Thomas S (2023). Birth plans: definitions, content, effects, and best practices. Am J Obstet Gynecol.

[13] Shareef N, Scholten N, Nieuwenhuijze M, Stramrood C, Vries M, van Dillen J. (2023). The role of birth plans for shared decision-making around birth choices of pregnant women in maternity care: a scoping review. Women Birth.

[14] Whittemore R, Knafl K. (2005). The integrative review: updated methodology. J Adv Nurs.

[15] Page MJ, McKenzie JE, Bossuyt PM, Boutron I, Hoffmann TC, Mulrow CD (2021). The PRISMA 2020 statement: an updated guideline for reporting systematic reviews. BMJ.

[16] Ouzzani M, Hammady H, Fedorowicz Z, Elmagarmid A. (2016). Rayyan-a web and mobile app for systematic reviews. Syst Rev.

[17] Melnyk BM, Fineout-Overholt E. (2005). Philadelphia.

[18] Ahmadpour P, Moosavi S, Mohammad-Alizadeh-Charandabi S, Jahanfar S, Mirghafourvand M. (2024). The childbirth experiences of Iranian women with birth plans. Heliyon.

[19] Artieta-Pinedo I, Paz-Pascual C, Garcia-Alvarez A, Bully P. (2024). Does the birth plan match what is relevant to women? preferences of Spanish women when giving birth. BMC Womens Health.

[20] Chantry AA, Merrer J, Blondel B, Le Ray C. (2023). Preferences for labor and childbirth, expressed orally or as a written birth plan: prevalence and determinants from a nationwide population-based study. Birth.

[21] Barnes C, Mignacca E, Mabbott K, Officer K, Hauck Y, Bradfield Z. (2023). Using a scheduled caesarean birth plan: a cross-sectional exploration of women’s perspectives. Women Birth.

[22] Guo H, Li T, Zhou R, Li M, Feng C, Cai X, Zhang C. (2023). The application of a continuous partnership-based birth plan in China: a randomized controlled trial. Midwifery.

[23] Alba-Rodríguez R, Coronado-Carvajal MP, Hidalgo-Lopezosa P. (2022). The Birth Plan Experience: a pilot qualitative study in Southern Spain. Healthcare (Basel).

[24] Ahmadpour P, Moosavi S, Mohammad-Alizadeh-Charandabi S, Jahanfar S, Mirghafourvand M. (2022). Effect of implementing a birth plan on maternal and neonatal outcomes: a randomized controlled trial. BMC Pregnancy Childbirth.

[25] López-Gimeno E, Seguranyes G, Vicente-Hernández M, Burgos Cubero L, Vázquez Garreta G, Falguera-Puig G. (2022). Effectiveness of birth plan counselling based on shared decision making: a cluster randomized controlled trial (APLANT). PLoS One.

[26] Hidalgo-Lopezosa P, Cubero-Luna AM, Jiménez-Ruz A, Hidalgo-Maestre M, Rodríguez-Borrego MA, López-Soto PJ. (2021). Association between Birth Plan Use and Maternal and Neonatal Outcomes in Southern Spain: a case-control study. Int J Environ Res Public Health.

[27] López-Gimeno E, Falguera-Puig G, Vicente-Hernández MM, Angelet M, Garreta GV, Seguranyes G. (2021). Birth plan presentation to hospitals and its relation to obstetric outcomes and selected pain relief methods during childbirth. BMC Pregnancy Childbirth.

[28] Jolles MW, Vries M, Hollander MH, van Dillen J. (2019). Prevalence, characteristics, and satisfaction of women with a birth plan in The Netherlands. Birth.

[29] Soriano-Vidal FJ, Vila-Candel R, Soriano-Martín PJ, Tejedor-Tornero A, Castro-Sánchez E. (2018). The effect of prenatal education classes on the birth expectations of Spanish women. Midwifery.

[30] Afshar Y, Mei JY, Gregory KD, Kilpatrick SJ, Esakoff TF. (2018). Birth plans-Impact on mode of delivery, obstetrical interventions, and birth experience satisfaction: a prospective cohort study. Birth.

[31] Westergren A, Edin K, Walsh D, Christianson M. (2019). Autonomous and dependent-The dichotomy of birth: a feminist analysis of birth plans in Sweden. Midwifery.

[32] Hidalgo-Lopezosa P, Hidalgo-Maestre M, Rodríguez-Borrego MA. (2017). Birth plan compliance and its relation to maternal and neonatal outcomes. Rev Latino-Am Enfermagem.

[33] Divall B, Spiby H, Nolan M, Slade P. (2017). Plans, preferences or going with the flow: an online exploration of women’s views and experiences of birth plans. Midwifery.

[34] Anderson CM, Monardo R, Soon R, Lum J, Tschann M, Kaneshiro B. (2017). Patient communication, satisfaction, and trust before and after use of a standardized birth plan. Hawaii J Med Public Health.

[35] Afshar Y, Wang ET, Mei J, Esakoff TF, Pisarska MD, Gregory KD. (2017). Childbirth education class and birth plans are associated with a vaginal delivery. Birth.

[36] Mouta RJO, Silva TMA, Melo PTS, Lopes NS, Moreira VA. (2017). Plano de Parto como estratégia de empoderamento feminino. Rev Baiana Enferm.

[37] Mei JY, Afshar Y, Gregory KD, Kilpatrick SJ, Esakoff TF. (2016). Birth plans: what matters for birth experience satisfaction. Birth.

[38] Suárez-Cortés M, Armero-Barranco D, Canteras-Jordana M, Martínez-Roche ME. (2015). Use and influence of Delivery and Birth Plans in the humanizing delivery process. Rev Latino-Am Enfermagem.

[39] Vila-Candel R, Mateu-Ciscar C, Bellvis-Vázquez E, Planells-López E, RequenaMarín M, Gómez-Sánchez MJ. (2015). Influencia del programa de educación maternal en el cambio de preferencias del plan de parto en gestantes del Departamento de Salud de La Ribera. Matronas Prof.

[40] Whitford HM, Entwistle VA, van Teijlingen E, Aitchison PE, Davidson T, Humphrey T, Tucker JS. (2014). Use of a birth plan within woman-held maternity records: a qualitative study with women and staff in northeast Scotland. Birth.

[41] Hidalgo-Lopezosa P, Rodríguez-Borrego MA, Muñoz-Villanueva MC. (2013). Are birth plans associated with improved maternal or neonatal outcomes?. MCN Am J Matern Child Nurs.

[42] Magoma M, Requejo J, Campbell O, Cousens S, Merialdi M, Filippi V. (2013). The effectiveness of birth plans in increasing use of skilled care at delivery and postnatal care in rural Tanzania: a cluster randomised trial. Trop Med Int Health.

[43] Hadar E, Raban O, Gal B, Yogev Y, Melamed N. (2012). Obstetrical outcome in women with self-prepared birth plan. J Matern Fetal Neonatal Med.

[44] Pennell A, Salo-Coombs V, Herring A, Spielman F, Fecho K. (2011). Anesthesia and analgesia-related preferences and outcomes of women who have birth plans. J Midwifery Womens Health.

[45] Sheridan CP, Yekinni I, Oyeye G, Ogunleye K, Oluyede G, O’Sullivan K (2011). Comparing birth plan preferences among Irish and Nigerian women. Brit J Midwifery.

[46] Sato S, Umeno Y. (2011). The relationship between the recognition of postpatum mothers’ birth plan and the degree of satisfaction with delivery. J Japan Acad Midwifery.

[47] Kuo SC, Lin KC, Hsu CH, Yang CC, Chang MY, Tsao CM, Lin LC. (2010). Evaluation of the effects of a birth plan on Taiwanese women’s childbirth experiences, control and expectations fulfilment: a randomised controlled trial. Int J Nurs Stud.

[48] Yam EA, Grossman AA, Goldman LA, García SG. (2007). Introducing birth plans in Mexico: an exploratory study in a hospital serving low-income Mexicans. Birth.

[49] Deering MA, Heller J, McGaha K, Heaton J, Satin AJ. (2006). Patients presenting with birth plans in a military tertiary care hospital: a descriptive study of plans and outcomes. Mil Med.

[50] Gulbrandsen P, Aarseth J, Aaby E, Valdal A. (2004). A birth plan effects and evaluation. Tidsskr Nor Laegeforen.

[51] Lundgren I, Berg M, Lindmark G. (2003). Is the childbirth experience improved by a birth plan?. J Midwifery Womens Health.

[52] Brown SJ, Lumley J. (1998). Communication and decision-making in labour: do birth plans make a difference?. Health Expect.

[53] Whitford HM, Hillan EM. (1998). Women’s perceptions of birth plans. Midwifery.

[54] Moore M, Hopper U. (1995). Do birth plans empower women? evaluation of a hospital birth plan. Birth.

[55] Smoleniec JS, James DK (1992). Does Having a Birth Plan Affect Operative Delivery Rate?. J Obstetr Gynaecol.

