# Supplementary material for: Use of birth plans by pregnant women and its impact on childbirth: an integrative review
Source: Rev Bras Enferm. 2026 Jun 22;79:e20250381. doi: 10.1590/0034-7167-2025-0381 (PMC13317011; doi:10.1590/0034-7167-2025-0381)
Supplement: Translation

# Translation: Uso do plano de parto por gestantes e seus impactos no parto: revisão integrativa

**DOI:** 10.1590/0034-7167-2025-0381pt

**Authors:** Paola Coutinho Barreto, Maria Luziene de Sousa Gomes, Roger Rodrigues da Silva, Eremita Val Rafael, Kayo Elmano Costa da Ponte Galvão, José de Ribamar Medeiros Lima, Claudia Teresa Frias Rios, Mônica Oliveira Batista Oriá

**Affiliations:** I Universidade Federal do Maranhão. São Luís, Maranhão, Brasil; II Universidade Federal do Ceará. Fortaleza, Ceará, Brasil

**Keywords:** Plano de Parto, Gestantes, Tomada de Decisão Compartilhada, Parto Humanizado, Promoção da Saúde.

## Abstract

**Objetivos:**

avaliar as evidências sobre o uso do plano de parto por gestantes.

**Métodos:**

revisão integrativa, realizada em sete bases de dados sem delimitação temporal e de idioma. A análise foi realizada de maneira qualitativa e descritiva.

**Resultados:**

foram incluídos 41 estudos. O uso do PP: facilita a comunicação entre gestantes e profissionais de saúde; promove a participação ativa da mulher; fortalece a autonomia; favorece a tomada de decisão compartilhada; e aumenta satisfação com o processo de parto. Verificou-se associação com redução de cesarianas, aumento de partos vaginais, aumento da pontuação de Apgar e maior probabilidade de início da amamentação. Os itens mais frequentes foram contato pele a pele, presença do acompanhante, métodos para alívio da dor, posição expulsiva, informação sobre intervenções médicas, ingestão hídrica e deambulação.**Conclusões:** o plano de parto é ferramenta essencial para humanizar a assistência obstétrica, devendo ser apresentado e discutido ainda no pré-natal.

## INTRODUÇÃO

O uso do plano de parto (PP) é recomendado pela Organização Mundial da Saúde (OMS)^(1)^, e no Brasil, pelo Ministério da Saúde, após a implantação do Programa Rede Cegonha, em 2011^(2)^. O PP viabiliza a apropriação de informações que promovem benefícios à autonomia e protagonismo das mulheres quanto à sensibilização dos profissionais de saúde que assistem-nas^(3,4)^. No PP, as gestantes expressam suas vontades e desejos durante o seu trabalho de parto (TP), parto e pós-parto, que incluem desde a ingesta alimentar e hídrica até a posição na hora de parir, uso de medicamentos e procedimentos sem real necessidade, orientações sobre tudo que irá ser realizado^(4)^.

Apesar dos benefícios da construção do PP e da sua comprovada eficácia, o desconhecimento quanto à sua existência e ao descumprimento dos desejos comunicados pelas das gestantes ainda é predominante. Enfermeiros e médicos devem incorporar o uso do PP em suas consultas, especialmente no pré-natal realizado na Atenção Primária à Saúde (APS), com o intuito de qualificar a assistência e fortalecer a comunicação entre a gestante e a equipe de saúde no ambiente hospitalar. Ademais, essa estratégia favorece o acesso à informação, a tomada de decisão informada e a construção de uma responsabilidade compartilhada entre o profissional de saúde e a mulher^(3)^.

O PP está dentro do passo um entre os “10 passos do Cuidado Obstétrico para Redução da Morbimortalidade Materna”, que aborda os encontros de qualidade centrados nas necessidades de cada mulher durante todos os contatos com os serviços de saúde, estimulando a discussão e elaboração conjunta do PP^(5)^. É por meio do PP que a equipe conhecerá os desejos e preferências das gestantes, contribuindo para que estes sejam alcançados e respeitados^(6)^.

Nesse sentido, o PP viabiliza para as mulheres a oportunidade de escolhas, que valorizam o respeito, a garantia de direitos, as relações humanizadas e as práticas baseadas em evidências científicas^(7)^. Envolver as mulheres no processo de tomada de decisão em seu TP por meio do PP pode ser considerado uma intervenção estratégica, que pode melhorar os resultados maternos e neonatais, além de oportunizar maior empoderamento^(8,9)^.

Este estudo surge com o propósito claro de aproximar o Objetivo de Desenvolvimento Sustentável 3 (saúde e bem-estar) à promoção do uso do PP, incentivando sua construção e utilização pelas mulheres para participação ativa no parto. Diante disso, o estudo se justifica para a prática da enfermagem, ao apresentar uma ferramenta voltada ao empoderamento e à autonomia feminina, além de contribuir para a divulgação do PP como um meio para favorecer o alcance das seguintes metas: meta 3.1: até 2030, reduzir a taxa de mortalidade materna global para menos de 70 mortes por 100.000 nascidos vivos; meta 3.7: assegurar o acesso universal aos serviços de saúde sexual e reprodutiva, incluindo o planejamento familiar, informação e educação, bem como a integração da saúde reprodutiva em estratégias e programas nacionais; e meta 5.6: assegurar o acesso universal à saúde sexual e reprodutiva e aos direitos reprodutivos^(10)^.

Revisões anteriores abordam o PP em aspectos como seu propósito, processo, impacto^(11)^, definições, conteúdo, efeitos e melhores práticas^(12)^, e seu papel na tomada de decisão compartilhada^(13)^. Não foi identificada revisão específica que sintetize as evidências sobre o uso do PP pelas gestantes e seus impactos no processo de parto. Diante dessa lacuna, este estudo tem como objetivo avaliar as evidências sobre o uso do PP por gestantes. Como objetivo secundário, busca-se analisar os impactos dessa utilização sobre o processo assistencial, a experiência materna e os desfechos do parto.

## OBJETIVOS

Avaliar as evidências sobre o uso do PP por gestantes.

## MÉTODOS

### Aspectos éticos

Este estudo foi baseado em dados que foram publicados e estão disponíveis publicamente; portanto, não requer submissão ou aprovação do Comitê de Ética em Pesquisa. Vale destacar que foram seguidos os princípios éticos fundamentais da pesquisa científica, incluindo a transparência metodológica, a integridade acadêmica e o respeito aos direitos autorais.

### Desenho do estudo

Trata-se de revisão integrativa da literatura, fundamentada no referencial teórico proposto por Whittemore e Knafl, realizada em cinco estágios: elaboração da questão norteadora; busca e seleção dos estudos primários; avaliação dos estudos primários; análise dos dados; e apresentação da revisão^(14)^. Esta estrutura metodológica foi adotada com o objetivo de assegurar uma abordagem sistemática na síntese das evidências. Pretendeu-se, com isso, obter uma compreensão abrangente sobre o uso do PP por gestantes, bem como identificar lacunas a serem exploradas em estudos futuros^(14)^. A redação do estudo seguiu as recomendações do *Preferred Reporting Items for Systematic Reviews and Meta-Analyses* (PRISMA)^(15)^.

### Questão norteadora

A questão norteadora definida para conduzir esta revisão integrativa foi: o que revelam as evidências científicas sobre a utilização do PP por gestantes? Para elaboração dessa questão, foi adotado o acrônimo PICo (População, Interesse e Contexto), sendo P =população (gestantes), I =interesse (PP) e Co =contexto (serviços de saúde).

### Critérios de elegibilidade

Foram incluídos estudos primários relacionados à temática, realizados com gestantes, sem restrição de idioma ou período de publicação. Optou-se por não estabelecer recorte temporal, pois o objetivo deste estudo foi reunir toda a literatura disponível sobre o uso do PP por gestantes. Tal decisão se justifica por se tratar da primeira revisão integrativa a sintetizar as evidências acerca do tema, contemplando desde as primeiras publicações até o período final da coleta. Foram excluídos trabalhos de conclusão de curso, dissertações, teses, anais de eventos e editoriais.

### Busca e seleção dos estudos

A busca dos estudos primários foi realizada em 17 de setembro de 2024 nas bases de dados *MEDical Literature Analysis and Retrieval System Online* (MEDLINE)/PubMed (via *National Library of Medicine*), *Web of Science*, Scopus, *Cumulative Index to Nursing and Allied Health Literature* (CINAHL da EBSCO), Cochrane, EMBASE (Elsevier) e Literatura Latino-Americana e do Caribe em Ciências da Saúde (LILACS) via Biblioteca Virtual em Saúde. As bases de dados foram acessadas gratuitamente por meio do Portal de Periódicos da Coordenação de Aperfeiçoamento de Pessoal de Nível Superior.

Os termos de busca selecionados no *Medical Subject Headings* foram aplicados na MEDLINE, *Web of Science*, Scopus, CINAHL, Cochrane, LILACS, e nos *Emtree Terms*, na *Excerpta Medica dataBASE* (EMBASE). As estratégias de busca foram elaboradas por meio da combinação de descritores e palavras-chave, a partir dos operadores booleanos *OR* e *AND*, de acordo com o Quadro 1.

**Quadro 1:** Estratégia de busca utilizada nas bases de dados, São Luís, Maranhão, Brasil, 2024

Base de dados	Estratégia de busca
MEDLINE/PubMed *Web of Science* Scopus CINAHL Cochrane EMBASE LILACS	(“*Pregnant women*” *OR Pregnancy OR Women*) *AND* (“*Birth plan*” *OR* “*Maternity care plan*”)

*MEDLINE - MEDical Literature Analysis and Retrieval System Online; CINAHL - Cumulative Index to Nursing and Allied Health Literature; EMBASE - Excerpta Medica dataBASE; LILACS - Literatura Latino-Americana e do Caribe em Ciências da Saúde.*

Os resultados identificados nas bases de dados foram exportados para o *software* Rayyan^(16)^. A seleção dos estudos foi realizada por dois revisores, de forma independente, em duas etapas, e seguiu as recomendações (identificação, triagem e inclusão) do fluxograma PRISMA^(15)^. Na primeira etapa, houve a leitura dos títulos e resumos e a aplicação dos critérios de elegibilidade. Posteriormente, os revisores se reuniram para discutir as disparidades na seleção e chegar a um consenso. Na etapa seguinte, realizou-se a leitura dos textos na íntegra, e foram aplicados, novamente, os critérios de elegibilidade. Situações de discordância, ao final da etapa, foram resolvidas com o parecer de um terceiro revisor. Ressalta-se que a busca manual na lista de referências dos estudos primários incluídos foi realizada com a finalidade de identificar evidências adicionais relacionadas à temática de interesse.

### Coleta de dados

A coleta de dados correspondente à caracterização dos estudos ocorreu mediante o desenvolvimento e utilização de formulário de extração de dados, sendo extraídas as seguintes variáveis: autoria; ano de publicação; país; objetivo do estudo; desenho do estudo; e principais resultados. A referida etapa foi realizada por dois revisores, de forma independente. Nos casos em que ocorreram divergências, foi realizada reunião para discussão até que ocorresse um consenso.

### Tratamento e análise de dados

Os resultados foram analisados e sintetizados de forma descritiva, utilizando uma abordagem de síntese narrativa. Primeiramente, os estudos foram agrupados para oferecer uma visão geral abrangente do escopo, da natureza e da distribuição dos estudos incluídos na revisão, apresentados em um quadro de caracterização. Para a classificação do nível de evidência (NE) dos estudos, foi utilizado modelo proposto por Melnyk e Fineout Overholt^(17)^_,_ o qual é dividido nos seguintes níveis: nível I - evidências oriundas de revisão sistemática ou metanálise de todos ensaios clínicos relevantes randomizados controlados ou provenientes de diretrizes clínicas baseadas em revisões sistemáticas de ensaios clínicos randomizados controlados; nível II - evidências derivadas de, pelo menos, um ensaio clínico randomizado controlado bem delineado; nível III - evidências obtidas de ensaios clínicos bem delineados sem randomização; nível IV - evidências provenientes de estudos de coorte e de caso-controle bem delineados; nível V - evidências originárias de revisão sistemática de estudos descritivos e qualitativos; nível VI - evidências derivadas de um único estudo descritivo ou qualitativo; e nível VII - evidências oriundas de opinião de autoridades e/ou relatórios de comitês de especialistas. Em seguida, os resultados foram discutidos com base em categorias temáticas identificadas pelos autores após a leitura dos estudos incluídos.

## RESULTADOS

No total, foram identificados 1.015 registros. Depois de remover as duplicatas, 369 foram deixados para triagem. Após triagem de títulos e resumos, 316 registros foram excluídos por não responderem aos critérios de inclusão, e cinco registros não foram recuperados, deixando 48 estudos potencialmente relevantes. Após leitura na íntegra, sete não atendiam aos critérios, restando 41 estudos (Figura 1).

**Figura 1:** Fluxograma *Preferred Reporting Items for Systematic Reviews and Meta-Analyses*: busca e seleção dos artigos incluídos, São Luís, Maranhão, Brasil, 2024

### Caracterização dos estudos incluídos

A síntese descritiva dos estudos primários conforme autoria, ano de publicação, país do estudo, tipo de estudo, amostra, cenário e NE é apresentada no Quadro 2.

**Quadro 2:** Síntese dos estudos incluídos na revisão integrativa (N=41), São Luís, Maranhão, Brasil, 2024

ID	Autor, (ano) e país	Objetivo	Tipo de estudo, amostra e cenário	NE
E1	Ahmadpour *et al*. (2024)^(18)^ Irã	Explorar e entender as perspectivas de mulheres que utilizaram um PP durante o parto.	Estudo: qualitativo Amostra: n=14 mulheres Cenário: Hospital Taleghani em Tabriz, Irã Setor de atuação: não especificado	VI
E2	Artieta-Pinedo *et al*. (2024)^(19)^ Espanha	Explorar quais das opções incluídas nos PPs são de maior interesse para as mulheres e quais são mais controversas.	Estudo: transversal Amostra: n=247 mulheres Cenário: 20 consultórios de parteiras Setor de atuação: não especificado	VI
E3	Chantry *et al*. (2023)^(20)^ França	Avaliar a prevalência de parturientes com preferências para seu TP e parto, expressas verbalmente na sala de parto ou como um PP escrito, e estudar fatores maternos, obstétricos e organizacionais associados à sua expressão.	Estudo: transversal Amostra: n=11.633 parturientes Cenário: 513 maternidades na França Setor de atuação: não informado	VI
E4	Barnes *et al*. (2023)^(21)^ Austrália	Explorar as experiências e perspectivas de mulheres que utilizaram um PP cesáreo programado em hospital de maternidade terciário australiano.	Estudo: transversal Amostra: n=294 participantes Cenário: hospital de maternidade terciário australiano Setor de atuação: não informado	VI
E5	Guo *et al*. (2023)^(22)^ China	Avaliar os efeitos de PP baseado em parceria contínua nos resultados e na experiência do parto de mulheres locais em Haikou, na China.	Estudo: ensaio clínico randomizado Amostra: n=90 mulheres Cenário: hospitais terciários na cidade de Haikou, província de Hainan, China Setor de atuação: clínica de obstetrícia dos hospitais	II
E6	Mohaghegh *et al*. (2023)^(8)^ Irã	Investigar o efeito do PP junto com as aulas de preparação pré-natal nos resultados maternos e neonatais.	Estudo: ensaio clínico randomizado Amostra: n=300 mulheres Cenário: quatro centros de saúde pública em Teerã, Irã + dois hospitais privados e um hospital afiliado às forças armadas Setor de atuação: não informado	II
E7	Alba-Rodríguez *et al*. (2022)^(23)^ Espanha	Determinar o impacto da apresentação de PP na experiência pessoal das mulheres, com foco em suas expectativas e no nível de satisfação alcançado.	Estudo: qualitativo Amostra: n=7 mulheres Cenário: sala de enfermagem do hospital terciário do Sistema Público de Saúde da Andaluzia (Espanha) Setor de atuação: sala de consulta de enfermagem em um centro de saúde	VI
8	Ahmadpour *et al*. (2022)^(24)^ Irã	Investigar o efeito do PP nos resultados maternos e neonatais.	Estudo: ensaio clínico randomizado Amostra: n=106 gestantes Cenário: hospital educacional Taleghani na cidade de Tabriz, Irã Setor de atuação: Clínica de Obstetrícia do Hospital de Pesquisa e Treinamento Médico Taleghani	II
9	López-Gimeno *et al*. (2022)^(25)^ Espanha	Identificar se mulheres que apresentaram PP no hospital têm melhores resultados obstétricos e ficam mais satisfeitas com a experiência do parto.	Estudo: ensaio clínico randomizado Amostra: n=461 gestantes Cenário: quatro Unidades de Atenção Primária do Sistema Nacional de Saúde da Catalunha (Espanha) Setor de atuação: não informado	II
10	Trigueiro *et al*. (2022)^(6)^ Brasil	Descrever a experiência das gestantes atendidas na consulta de enfermagem a partir de 37 semanas e que elaboraram seu PP.	Estudo: qualitativo Amostra: n=19 gestantes Cenário: maternidade de risco habitual em Curitiba Setor de atuação: ambulatório da maternidade	VI
11	Hidalgo-Lopezosa *et al*. (2021)^(26)^ Espanha	Comparar resultados obstétricos e neonatais entre mulheres com e sem PP.	Estudo: caso-controle retrospectivo Amostra: n=457 gestantes Cenário: hospitais terciários no sul da Espanha Setor de atuação: quatro hospitais públicos terciários com a maior cobertura em cada província	IV
12	López-Gimeno *et al*. (2021)^(27)^ Espanha	Identificar a porcentagem de gestantes que apresentaram PP em cinco hospitais na Espanha, os motivos pelos quais algumas mulheres não o fizeram e como a apresentação do PP se relaciona com os resultados obstétricos e os métodos selecionados de alívio da dor.	Estudo: descritivo e multicêntrico Amostra: n=422 mulheres Cenário: centros de saúde primários em vários distritos de saúde em Barcelona (Catalunha, Espanha) Setor de atuação: cinco unidades Assistência à Saúde Sexual e Reprodutiva do Instituto Catalão de Saúde em centros de atenção primária localizados na Catalunha	VI
13	Loiola *et al*. (2020)^(7)^ Brasil	Analisar a percepção de mulheres que utilizaram o PP em uma casa de parto do sudeste do Brasil.	Estudo: descritivo com abordagem qualitativa Amostra: n=11 puérperas Cenário: Casa de Parto do Rio de Janeiro Setor de atuação: consultórios de acompanhamento de pré-natal	VI
14	Jolles *et al*. (2019)^(28)^ Holanda	Avaliar a taxa de PP, características das mulheres e pontuações de satisfação pós-parto em um único centro na Holanda.	Estudo: retrospectivo Amostra: n=1.159 mulheres Cenário: *Amalia Children’s Hospital*, Radboudumc, Nijmegen Setor de atuação: hospital acadêmico	VI
15	Soriano-Vidal *et al*. (2018)^(29)^ Espanha	Avalia a influência de aulas educacionais pré-natais lideradas por parteiras sobre as preferências das mulheres em relação ao parto.	Estudo: multicêntrico, observacional e prospectivo Amostra: n=212 gestantes Cenário: centros de saúde de referência no sudeste da Espanha Setor de atuação: departamentos de saúde de La Ribera (março a setembro) e Xàtiva-Ontinyent (janeiro a setembro)	VI
16	Afshar *et al*. (2018)^(30)^ Estados Unidos	Examinar se a presença de um PP estava associada ao modo de parto, às intervenções obstétricas e à satisfação da paciente.	Estudo: coorte prospectivo Amostra: n=300 mulheres Cenário: grande hospital terciário urbano em Los Angeles Setor de atuação: centros de parto e enfermaria de recuperação pós-parto	IV
17	Westergren *et al*. (2019)^(31)^ Suécia	Evocar as percepções das mulheres grávidas sobre o parto, expressas em seus PP e, por meio de uma lente feminista, analisar seus desejos, medos, valores e crenças sobre o parto, bem como suas expectativas em relação ao parceiro e à parteira.	Estudo: qualitativo Amostra: n=132 mulheres Cenário: uma cidade de médio porte no norte da Suécia Setor de atuação: *online:* por meio de registros médicos eletrônicos pré-natais e intraparto	VI
18	Hidalgo-Lopezosa *et al*. (2017)^(32)^ Espanha	Conhecer o grau de cumprimento das solicitações que as mulheres registram nos seus PP e determinar sua influência nos principais resultados obstétricos e neonatais.	Estudo: retrospectivo, descritivo e analítico Amostra: n=178 mulheres com PP Cenário: hospital de nível terciário do Sistema Público de Saúde da Andaluzia Setor de atuação: não especificado	VI
19	Divall *et al*. (2017)^(33)^ Reino Unido	Explorar as opiniões das mulheres sobre os PP e as experiências de sua conclusão e utilização.	Estudo: qualitativo Amostra: não especificada Cenário: *online* Setor de atuação: não especificado	VI
20	Anderson *et al*. (2017)^(34)^ Havaí	Descrever como um grupo de mulheres cultural e educacionalmente diversas que se apresentaram para atendimento obstétrico em Honolulu, Havaí, classificaram a comunicação, a satisfação e a confiança com o uso de um PP padronizado durante o TP e parto.	Estudo: projeto de melhoria da qualidade Amostra: n=81 mulheres Cenário: Clínica Ambulatorial Feminina do *Kapi‘olani Medical Center* em Honolulu, Havaí Setor de atuação: não informado	VI
21	Afshar *et al*. (2017)^(35)^ Estados Unidos	Determinar se o modo de parto foi diferente entre mulheres que frequentaram aulas de educação para o parto, tiveram um PP ou ambos, em comparação com aquelas que não frequentaram aulas ou tiveram um PP.	Estudo: transversal retrospectivo Amostra: n=14.630 mulheres Cenário: hospital terciário urbano em Los Angeles Setor de atuação: centro de atendimento terciário urbano	VI
22	Mouta *et al*. (2017)^(36)^ Brasil	Analisar como o PP propiciou o empoderamento feminino durante o TP e parto.	Estudo: exploratório qualitativo Amostra: n=11 puérperas Cenário: Casa de Parto localizada no município do Rio de Janeiro, Brasil Setor de atuação: não informado	VI
23	Mei *et al*. (2016)^(37)^ Estados Unidos	Categorizar solicitações individuais de PP e determinar se o número de solicitações e o atendimento das solicitações estão associados à satisfação com a experiência do parto.	Estudo: coorte prospectivo Amostra: n=109 mulheres Cenário: um grande centro médico urbano de cuidados terciários em Los Angeles Setor de atuação: não informado	IV
24	Suárez-Cortés *et al*. (2015)^(38)^ Espanha	Conhecer, analisar e descrever a situação atual dos PPs e nascimento no contexto estudado, comparando o processo de parto e sua finalização entre as mulheres que apresentaram e as que não apresentaram um PP e nascimento.	Estudo: coorte quantitativo, transversal, observacional, descritivo e comparativo Amostra: n=9.303 mulheres Cenário: Hospital Clínico Universitário Virgen de la Arrixaca de Murcia Setor de atuação: sala de parto	IV
25	Vila-Candel *et al*. (2015)^(39)^ Espanha	Verificar se há diferenças entre as preferências que refletem as gestantes no PP entre antes e depois de receber as sessões de educação materna.	Estudo: epidemiológico, observacional, longitudinal e prospectivo Amostra: n=249 gestantes Cenário: Departamento de Saúde de La Ribera da Agência Valenciana de Saúde Setor de atuação: centros de atenção primária do Departamento de Saúde de La Ribera	VI
26	Whitford *et al*. (2014)^(40)^ Escócia	Considerar o uso de uma seção de PP padrão dentro de um registro nacional de maternidade mantido por mulheres.	Estudo: exploratório, qualitativo e longitudinal Amostra: n=42 mulheres Cenário: duas regiões do *National Health Service Board* no nordeste da Escócia Setor de atuação: as entrevistas foram realizadas nas casas das mulheres ou nas dependências da universidade ou do serviço de saúde	VI
27	Hidalgo Lopesoza *et al*. (2013)^(41)^ Espanha	Determinar se os planos de parto estão associados a melhores resultados obstétricos e neonatais.	Estudo: retrospectivo de caso-controle Amostra: n=182 mulheres Cenário: hospital em Córdoba, Espanha Setor de atuação: não informado	IV
28	Magoma *et al.* (2013)^(42)^ Tanzânia	Determinar a eficácia dos PPs no aumento do uso de cuidados especializados no parto e no período pós-natal entre participantes de cuidados pré-natais em um distrito rural.	Estudo: ensaio randomizado por *cluster* Amostra: n=900 mulheres Cenário: distrito de Ngorongoro, região de Arusha Setor de atuação: 16 unidades de saúde e, posteriormente, casas das mulheres na aldeia no distrito para a entrevista pós-natal	II
29	Hadar *et al*. (2012)^(43)^ Israel	Determinar se a introdução de um PP pré-preparado na admissão em TP tem impacto no resultado obstétrico.	Estudo: retrospectivo Amostra: n=154 mulheres foram comparadas a um grupo controle pareado de 462 mulheres Cenário: centro médico terciário em Israel Setor de atuação: não informado	VI
30	Pennell *et al*. (2011)^(44)^ Estados Unidos	Descrever as preferências e os resultados relacionados à anestesia e analgesia de mulheres que usaram um PP para o TP e o parto.	Estudo: coorte prospectivo Amostra: n=63 Cenário: hospital universitário de atendimento terciário nos Estados Unidos Setor de atuação: não informado	IV
31	Sheridan *et al*. (2011)^(45)^ Irlanda	Comparar as preferências de PP entre mulheres grávidas irlandesas e nigerianas.	Estudo: observacional Amostra: n=632 mulheres Cenário: *Unified Maternity Services* em Cork, Irlanda Setor de atuação: clínica pré-natal de reserva	VI
32	Sato; Umeno (2011)^(46)^ Japão	Investigar o conhecimento dos PPs entre as mulheres grávidas e esclarecer a relação entre o reconhecimento das puérperas de seu PP e o grau de satisfação com seu parto, bem como examinar o papel da parteira em dar satisfação com o parto.	Estudo: pesquisa por questionário Amostra: n=442 puérperas Cenário: três clínicas obstétricas da prefeitura Setor de atuação: não especificado	VI
33	Kuo *et al*. (2010)^(47)^ China	Avaliar os efeitos dos PPs no cumprimento das expectativas das mulheres em relação ao parto, seu controle sobre o processo de parto e suas experiências gerais.	Estudo: ensaio clínico randomizado, simples-cego e controlado Amostra: n=296 gestantes Cenário: sete hospitais em Taiwan Setor de atuação: unidades médicas na parte norte e central de Taiwan	II
34	Yam *et al*. (2007)^(48)^ México	Avaliar a viabilidade e aceitabilidade da introdução de PP em um hospital que atende mexicanos de baixo *status* socioeconômico e para documentar as perspectivas de mulheres e profissionais de saúde sobre as vantagens e barreiras na implementação de um programa de PP.	Estudo: exploratório Amostra: n=9 gestantes Cenário: *Hospital de la Familia* na Ciudad Juárez, México. Setor de atuação: não informado	VI
35	Deering *et al*. (2006)^(49)^ Estados Unidos	Avaliar que tipo de paciente prepara um PP, identificar solicitações comuns feitas e determinar o quão rigorosamente elas são seguidas durante o TP.	Estudo: descritivo Amostra: n=67 mulheres Cenário: hospital de treinamento militar *National Naval Medical Center* (Bethesda, Maryland) Setor de atuação: unidade de parto e parto do *National Naval Medical Center*	VI
36	Gulbrandsen *et al*. (2004)^(50)^ Noruega	Descobrir se a utilização do PP contribuiu para proporcionar às mulheres uma melhor experiência e segurança durante a gravidez, parto e maternidade, e descrever as experiências das mulheres na utilização de tal formulário.	Estudo: pesquisa por questionário Amostra: n=300 gestantes Cenário: centros de saúde dos municípios de Eidsvoll, Fet, Gjerdrum, Rælingen e Ullensaker Setor de atuação: Hospital Universitário de Akershus	VI
37	Lundgren *et al*. (2003)^(51)^ Suécia	Avaliar os efeitos de responder a um questionário e formular um PP no final da gravidez nas experiências de parto das mulheres.	Estudo: pesquisa por questionário Amostra: n=271 mulheres Cenário: clínicas pré-natais na Suécia Setor de atuação: enfermaria de parto normal e na enfermaria de parto especial	VI
38	Brown; Lumley (1998)^(52)^ Austrália	Avaliar o uso de PP e examinar as diferenças nas características sociais e obstétricas, e as experiências intraparto de mulheres que usaram e não usaram um PP.	Estudo: pesquisa populacional Amostra: n=1.336 mulheres Cenário: 127 hospitais em Vitória, Austrália Setor de atuação: não informado	VI
39	Whitford; Hillan (1998)^(53)^ Escócia	Investigar o uso e os efeitos dos PP e como as mulheres os percebem.	Estudo: retrospectivo Amostra: n=143 primigestas Cenário: hospital universitário com aproximadamente 3.000 partos por ano em Dundee, Escócia Setor de atuação: não informado	VI
40	Moore; Hopper (1995)^(54)^ Austrália	Avaliar a facilidade de uso e eficácia da introdução do PP em dois hospitais.	Estudo: descritivo Amostra: n=100 mulheres Cenário: em dois hospitais distritais no sudoeste de Sydney (Austrália) Setor de atuação: clínicas hospitalares para cuidados pré-natais	VI
41	Smoleniec; James (1992)^(55)^ Inglaterra	Testar a hipótese de que ter um PP para o TP estava associado a um risco aumentado de parto operatório.	Estudo: descritivo Amostra: n=124 mulheres Cenário: não informado Setor de atuação: não informado	VI

*ID - identificação; NE - nível de evidência; PP - plano de parto; TP - trabalho de parto.*

No que se refere ao recorte temporal, as publicações incluídas datam do período de 1992 a 2024. O ano de 2017 (n=5) concentrou a maior parte das produções analisadas. A maioria dos estudos foi conduzida na Espanha^(19,23,25-27,29,32,38,39,41)^ e nos Estados Unidos^(30,35,37,44,49)^. Em relação à abordagem metodológica, os estudos se caracterizaram em sua maioria como qualitativos^(6,7,18,23,31,33,40)^, seguidos de ensaios clínicos^(8,22,24,25,42,47)^. A amostra dos estudos variou entre 247^(19)^ e 14.630 mulheres^(35)^. Os cenários de realização foram diversos, incluindo o ambiente hospitalar^(8,18,20-22,24,26,28,30,32,35,37,38,41,43-45,47-49,51-54)^, atenção básica^(25,27,29,39,42)^, ambulatório^(6,7,19,23,34,46)^, casa de parto^(7,36)^ e *online* ^(31,33)^. Quanto ao NE, a maioria dos estudos foi classificada como nível VI^(6,7,18-21,23,27-29,31-36,39,40,43,45,46,48-55)^.

A amostra do presente estudo, a partir dos artigos analisados, está demonstrada de forma sintetizada no material suplementar. Diante disso, a análise crítica e a síntese dos estudos selecionados foram realizadas de forma qualitativa (Quadro 3), emergindo a categorização de sete temas principais: conhecimento do PP; uso do PP; itens do PP; efeito do PP nos resultados maternos; efeito do PP nos resultados neonatais; satisfação e cumprimento do PP; e PP no pré-natal.

**Quadro 3:** Síntese dos proponentes do uso de plano de parto por gestantes, São Luís, Maranhão, Brasil, 2024

Conhecimento do PP	Desconhecimento do PP^(36)^ Desconhece ou conhece de forma superficial^(6)^ Nunca tinham ouvido falar de um PP anteriormente^(48)^
**Uso do PP**	Melhora a experiência geral do parto^(18,44)^ Facilita a comunicação^(11,13,18,21,23,24,33,34,40,44,47,48,54)^ Promove a participação ativa das mulheres durante o parto^(8,18,47)^ Oferece preparação física e mental^(18,52)^ Promove o empoderamento e autonomia das mulheres^(6,7,9,13,36)^ Facilita a tomada de decisão compartilhada^(13)^ e autonomia e controle^(11,13,24,33,44,47)^
**Itens do PP**	Contato pele a pele^(8,9,19,21,23,25,27,29,38,39,45,48)^ Presença do acompanhante^(19,31,45)^ ou pessoa de apoio^(21)^ Método de alívio da dor por métodos farmacológicos e não farmacológicos^(7,9,18,19,25,27,44,52)^ Informação e controle sobre a intervenção médica^(19,22,26)^ Posição expulsiva^(29,38,39,45)^ Caminhar durante o TP^(18,49)^ Uso da epidural^(27,45)^ Ingestão hídrica^(29,38,39)^ Ingestão de alimentos^(38)^ Abaixamento da tela cirúrgica^(20)^
**Efeito do PP nos resultados maternos**	Promove a redução de cesáreas e um maior índice de partos vaginais^(8,9,18,24,32,34,43,48,49,52)^ Reduz a intervenção médica^(8,22,26,32,37,40)^ Melhorar os resultados do parto^(21)^ Reduz a ansiedade^(6,8,22,32,40)^ Reduz o medo^(8,13,18,24,51)^ Reduz os sintomas psicológicos de depressão e TEPT^(23)^ Otimiza a experiência do parto^(21)^
**Efeito do PP nos resultados neonatais**	Aumento das pontuações de Apgar neonatais no primeiro minuto^(9,24,26,32,44)^ Clampeamento tardio do cordão^(38)^ Melhores resultados em pH do cordão umbilical^(9,26,32)^ Maior probabilidade de iniciar a amamentação na sala de parto^(8,19,23,27,29,37,39,48)^ Menor propensão a ter um RN internado na UTIN^(30,34,35)^
**Satisfação e cumprimento do PP**	Mais satisfação com o processo de parto^(8,9,11,18,24,28,34,46-48)^ Oportuniza uma melhor experiência, levando em consideração as preferências das mulheres^(23,52)^ O suporte e a comunicação durante o TP aumentam a satisfação da mulher com o parto^(24)^ Resultados de parto geralmente positivos^(9,11,50)^ Possibilita liberdade e autonomia no parir, sentindo-se ativas, respeitadas como mulher em seus desejos^(7)^ Mulheres com um PP e um parto vaginal espontâneo deram mais frequentemente uma pontuação alta para satisfação pós-parto^(28)^ O alto cumprimento do PP diminuiu a porcentagem de cesáreas e seus filhos melhores resultados neonatais^(32)^ O cumprimento das expectativas de parto garantiu um grau maior de domínio e participação das mulheres^(47)^ Maiores solicitações atendidas se correlacionaram com maior satisfação geral^(37)^ Mulheres que sabiam que a equipe tinha olhado para o seu PP durante o TP apreciaram que suas preferências foram seguidas ou pelo menos discutidas^(40)^ O PP influencia positivamente o TP e sua finalização, aumentando as dimensões de segurança, eficácia e satisfação das mulheres, assim como seu empoderamento^(39)^
**PP no pré-natal**	Ferramenta de educação^(13)^ Influencia as preferências das mulheres sobre o PP^(29,39)^ Maior expressão de preferências escritas ou verbais^(20)^ Influencia uma experiência positiva de parto^(18)^ Mulheres que realizaram o PP encorajariam outras mulheres a também usar^(54)^ Permite a expressão das necessidades e preferências, aumentando a confiança, e melhora a comunicação entre a equipe^(54)^ A consulta de enfermagem possibilitou a construção do PP^(6)^ Promove a participação das mulheres na tomada de decisão^(8,13)^ Favorece o cuidado obstétrico individualizado, garantindo o respeito às suas escolhas^(13)^ Reduz o medo e ansiedade^(8,13)^ Possibilita a obtenção de conhecimentos^(7)^ Aborda as necessidades individuais de suas pacientes^(42)^

*PP - plano de parto; TP - trabalho de parto; TEPT - transtorno de estresse pós-traumático; RN - recém-nascido; UTIN - unidade de terapia intensiva neonatal.*

## DISCUSSÃO

O conjunto de evidências analisadas permitiu compreender os fatores que influenciam o uso do PP por mulheres em diferentes contextos ao redor do mundo. Os estudos destacam achados notáveis sobre sua utilização, como facilitação da comunicação, promoção da participação ativa da mulher durante o parto, fortalecimento da autonomia e do controle sobre o próprio corpo, além de favorecer a tomada de decisão compartilhada e aumentar a satisfação com o processo de parto.

Além disso, o PP está associado à redução do número de cesáreas, ao aumento da taxa de partos vaginais, à diminuição do medo e da ansiedade, ao aumento da pontuação de Apgar no primeiro minuto de vida do recém-nascido (RN) e a uma maior probabilidade de início da amamentação na sala de parto. Outro benefício relevante é a menor propensão do RN à internação neonatal em Unidades de Terapia Intensiva. Entre os itens mais frequentes nos PPs, destacam-se contato pele a pele, presença do acompanhante, adoção de métodos para alívio da dor, escolha da posição expulsiva, obtenção de informação e controle sobre intervenções médicas, assim como possibilidade de ingestão hídrica e deambulação durante o TP.

O PP está entre as técnicas que devem ser estimuladas durante a gestação, de acordo com as normas internacionais preconizadas pela OMS, porém, em alguns serviços de saúde que atendem gestantes e parturientes, o PP ainda é pouco estimulado^(36)^. Apesar de ser um direito das gestantes, PP ainda é pouco conhecido ou compreendido, de forma superficial, por muitas mulheres em diversas regiões, o que impacta negativamente sua utilização. Estudo realizado no Paraná com 13 puérperas apontou que 69% delas não conheciam o que era o PP, sendo que 77% sequer imaginavam se tratar de um direito legal^(4)^. De maneira semelhante, em outro estudo realizado no Rio de Janeiro, apenas uma entre as 11 puérperas entrevistadas relatou conhecer o PP, enquanto todas as demais negaram qualquer familiaridade com o instrumento^(36)^. Da mesma forma, pesquisa conduzida em uma maternidade pública de Curitiba com 19 gestantes constatou que 13 delas desconheciam o PP ou haviam apenas ouvido falar, sem saber exatamente do que se tratava, reafirmando a carência de conhecimento mais aprofundado sobre o tema^(6)^.

Percebe-se que apenas uma pequena parcela das mulheres é informada sobre o PP por profissionais de saúde. Em pesquisa realizada em Curitiba, Paraná, constatou-se que outras fontes, como redes de apoio e a internet, foram os principais meios de acesso a informações sobre o PP e sobre temas relacionados à gestação e ao parto, evidenciando a necessidade de maior engajamento dos profissionais de saúde na divulgação e orientação sobre esse instrumento^(6)^. De forma semelhante, estudo conduzido na Andaluzia, Espanha, revelou que, embora a maioria das mulheres tenha valorizado as aulas de pré-natal, elas relataram a necessidade de que as equipes estejam atualizadas e que as aulas sejam melhor coordenadas. Apesar de terem sido informadas sobre a existência do PP, declararam dificuldades para obter informações detalhadas a respeito, recorrendo a outras fontes, como internet, livros, associações, familiares e amigos para se orientar^(23)^. Esses achados reforçam a importância de que os profissionais de saúde sejam os principais interlocutores na promoção e esclarecimento do PP, garantindo às mulheres acesso qualificado e confiável a essa ferramenta.

O PP, embora reconhecido legalmente, ainda apresenta baixa difusão entre profissionais de saúde, hospitais e maternidades, o que compromete sua aplicação prática^(36)^. Poucos serviços conseguem implementá-lo efetivamente, e muitos profissionais sequer conhecem seu significado^(3)^. Entre as barreiras institucionais e socioculturais, destacam-se o desconhecimento e a compreensão inadequada do PP por parte dos profissionais de saúde, a heterogeneidade dos próprios planos, e a falta de comunicação eficaz entre gestantes e equipe assistencial, o que gera percepções distorcidas de escolha e limita a autonomia da mulher^(6,11)^. O desconhecimento das gestantes sobre o processo de parto e sobre o próprio PP também contribui para dúvidas, medos e inseguranças^(6)^. Ademais, planos elaborados sem discussão prévia com os provedores de cuidado podem gerar expectativas irreais e insatisfação, sobretudo em contextos obstétricos altamente medicalizados, refletindo em perda de autonomia durante o parto^(11)^.

Para superar esses desafios, é fundamental que os serviços de saúde adotem estratégias que priorizem a comunicação e tomada de decisão compartilhada. O PP deve ser valorizado como uma ferramenta que facilita o diálogo, fortalece a autonomia da mulher e favorece práticas obstétricas mais humanizadas^(11,36)^. A elaboração colaborativa, com envolvimento ativo dos profissionais de saúde, é crucial para alinhar expectativas, otimizar resultados obstétricos, aumentar a satisfação e ampliar o senso de controle da gestante^(11)^. Nesse sentido, a consulta de enfermagem é configurada como um espaço estratégico para educação em saúde, possibilitando a utilização do PP como recurso educativo eficaz para esclarecer dúvidas, reduzir a ansiedade, promover o empoderamento da gestante e de seu acompanhante, e estabelecer vínculo com a maternidade, além de oferecer orientações claras sobre TP, parto e puerpério^(6,36)^.

Adicionalmente, recomenda-se a adoção de estratégias estruturais e organizacionais, como integração do PP aos protocolos de atenção pré-natal, capacitação multiprofissional voltada à assistência centrada na mulher e uso de ferramentas digitais que facilitem a elaboração, o registro e o compartilhamento do documento entre gestante e equipe de saúde^(3,4,6,11,23)^. Os profissionais de saúde desempenham um papel fundamental na concretização do PP, especialmente os enfermeiros, cuja atuação se inicia no pré-natal, momento em que o PP deve ser apresentado, e sua construção, estimulada. Durante o pré-natal, as gestantes têm a oportunidade de esclarecer dúvidas e contar com o apoio dos enfermeiros na elaboração do documento, fortalecendo a compreensão sobre o parto e o nascimento, e favorecendo a expressão de seus desejos, geralmente voltados à humanização e vivência natural do processo^(36)^. A elaboração do PP é validada como uma importante ferramenta de comunicação entre gestantes e profissionais de saúde, embora ainda seja necessário ampliar o envolvimento e a capacitação das equipes para que estejam mais conscientes das questões relacionadas ao respeito às escolhas das mulheres^(23)^.

Estudo qualitativo realizado no Irã identificou a preparação pré-natal como um fator-chave para uma experiência positiva de parto, destacando que a implementação do PP foi fundamental para aumentar a conscientização das gestantes, promover a prontidão física e mental, e capacitá-las a fazer escolhas informadas sobre métodos seguros de parto^(18)^. Achados semelhantes foram observados em pesquisa realizada no Paraná, em que 13 participantes relataram que as consultas pré-natais reduziram sua ansiedade e medo, proporcionando maior tranquilidade, segurança e confiança para o parto, tanto em relação aos aspectos fisiológicos quanto em relação às rotinas da maternidade^(6)^. Esses resultados reforçam a importância da educação pré-natal e do envolvimento ativo das mulheres no processo de tomada de decisão durante o parto. Aulas educativas e a construção do PP constituem estratégias fundamentais para preparar física e psicologicamente as gestantes, além de oferecer informações e alternativas para o manejo do TP^(8)^.

Em relação aos itens abordados no PP, estudo descritivo realizado na Espanha destacou que a maioria das mulheres considera essencial a presença do parceiro durante o parto, manutenção de um ambiente íntimo, contato contínuo com o bebê desde o nascimento e fornecimento de informações com solicitação de consentimento antes de qualquer intervenção^(19)^. De forma complementar, revisão narrativa apontou que mulheres com PP apresentaram maior probabilidade de iniciar a amamentação ainda na sala de parto. Embora a analgesia epidural tenha sido o método mais utilizado para alívio da dor, essas mulheres demonstraram maior propensão ao uso combinado de métodos não farmacológicos^(9)^.

Outro fator determinante para o desejo de elaboração e uso do PP é o grau de cumprimento de suas diretrizes. Estudo realizado com 178 mulheres na Andaluzia, Espanha, revelou que apenas 37% das gestantes que apresentaram o PP tiveram suas preferências majoritariamente respeitadas. Além disso, observou-se que quanto maior o nível de cumprimento do plano, melhores foram os desfechos maternos e neonatais^(32)^. Entre os principais motivos para o não cumprimento do PP, destaca-se a imprevisibilidade do processo de parto, como a ocorrência de distócias ou eventos inesperados que exigem mudanças nas condutas inicialmente planejadas^(32)^.

Embora o PP represente uma importante ferramenta de planejamento e comunicação, ele não garante que o TP e o nascimento ocorrerão conforme o desejado. Isso reforça a importância de gerenciar expectativas e promover uma comunicação clara entre profissionais de saúde e gestantes sobre as possíveis variações do processo^(18)^.

Para que o PP se consolide como uma ferramenta efetiva de cuidado, é necessário que sua implementação seja discutida com a gestão dos serviços de saúde e construída com o envolvimento ativo dos profissionais que atuam nesses contextos. O documento deve ser adaptado à realidade local, amplamente discutido com gestantes e acompanhantes e atualizado continuamente, garantindo que reflita as necessidades individuais, e ofereça experiências de parto mais positivas e seguras para todos os envolvidos^(6)^.

### Limitações do estudo

As limitações deste estudo estão relacionadas ao fato de que alguns estudos incluídos apresentaram fragilidades na descrição metodológica, como a ausência de especificação do tipo de estudo, ano de realização e tamanho da amostra. Observou-se ainda a predominância de pesquisas conduzidas nos Estados Unidos e na Espanha, com escassa produção nacional sobre o PP. Ademais, a maioria dos estudos incluídos corresponde ao nível VI de evidência, predominantemente qualitativos e descritivos, o que implica menor robustez metodológica, restringindo a generalização dos achados para diferentes contextos. Apesar dessas limitações, buscou-se seguir criteriosamente as recomendações para o desenvolvimento de revisões integrativas. Já os pontos fortes da pesquisa são que se trata de pesquisa nova, que fornece informações sobre o uso PP, com facilidade na busca e análise de conteúdos em estudos de vários países, por meio de livre acesso em plataformas didáticas com dados concretos para sua elaboração, sem custo financeiro ou necessidade de autorização do comitê de ética e pesquisa.

### Contribuições para as áreas da enfermagem, saúde ou políticas públicas

Os achados desta revisão ampliam o conhecimento sobre a utilização do PP em diversas regiões do mundo, oferecendo suporte para criação de materiais educativos que podem ser utilizados na prática clínica e em estudos futuros. Além disso, os resultados contribuem para disseminação do conhecimento entre profissionais, promovendo a valorização do PP como ferramenta para o cuidado centrado na gestante e tomada de decisão. Essa abordagem também estimula a pesquisa contínua sobre o tema, fortalecendo as práticas baseadas em evidências na área da saúde materna.

## CONCLUSÕES

Os estudos analisados indicam que o uso do PP facilita a comunicação entre gestantes e profissionais de saúde, promove a participação ativa da mulher, fortalece sua autonomia, e favorece a tomada de decisão compartilhada, resultando em maior satisfação com o processo de parto. Além disso, o PP se associa a melhores desfechos maternos e neonatais. Entre os itens mais recorrentes relatados pelas gestantes, destacam-se contato pele a pele, presença do acompanhante, métodos de alívio da dor, escolha da posição expulsiva, informações sobre intervenções médicas, ingestão hídrica e deambulação durante o TP.

Os resultados desta revisão reforçam que o PP é uma ferramenta fundamental para promoção de uma assistência obstétrica mais humanizada e centrada nas necessidades da mulher. Sua apresentação e incentivo durante o pré-natal são estratégicos para fortalecer a autonomia das gestantes, qualificar o cuidado prestado e aprimorar a experiência do parto. Esta revisão contribui para o aprofundamento do conhecimento sobre o uso do PP no contexto mundial. Sugere-se a realização de novas investigações que explorem o conhecimento e a utilização do PP no cenário brasileiro, especialmente na APS, onde se desenvolve o pré-natal e ocorre o primeiro contato da gestante com os serviços de saúde.

## Data Availability

Os dados de pesquisa estão disponíveis no corpo do artigo.
